# Lifestyle Practices and Obesity in Nepalese Youth: A Cross-sectional Study

**DOI:** 10.7759/cureus.2209

**Published:** 2018-02-20

**Authors:** Gaurav Nepal, Eans T Tuladhar, Saurav Dahal, Shaik Tanveer Ahamad, Sumikshya Adhikari, Apsara Kandel

**Affiliations:** 1 Maharajgunj Medical Campus, Tribhuvan University Institute of Medicine; 2 Department of Medicine, Deccan College of Medical Sciences; 3 Maharajgunj Nursing Campus, Tribhuvan University Institute of Medicine

**Keywords:** nepal, youth, lifestyle, obesity

## Abstract

Introduction

Understanding the lifestyle factors associated with obesity is critical to create a successful intervention that would prevent or reduce the obesity beforehand. However, these factors have not been assessed among Nepalese youths thus far. This study aims to determine the prevalence of obesity and to explore the potential lifestyle risk factors in young university students of Nepal.

Methods

We included in the study 384 young students aged between 17 and 24 years, pursuing medicine at Tribhuvan University, Institute of Medicine, in this cross-sectional study. A self-administered questionnaire to collect information about age, sex, smoking, alcohol consumption, meat consumption, fast-food consumption, and sedentary lifestyle was employed. Anthropometric measurements were taken to calculate body mass index (BMI) and waist-to-hip ratio (WHR).

Results

This study revealed that the current, episodic heavy alcohol consumers, current cigarette smokers, and individuals with a sedentary lifestyle had a statistically significant higher BMI and WHR as compared to age and gender-matched healthy subjects. Meat consumers as well had a statistically significant higher BMI. However, there has been no statistically significant difference in BMI and WHR in those who consume fast food from those who don’t.

Conclusion

Our study shows a high prevalence of obesity among young university students of Nepal, making it necessary to develop effective preventive measures to reduce their exposure to the risk factors associated with obesity. Early interventions to encourage lifestyle changes can be a worthwhile and effective strategy to prevent and/or reduce the risks for the development of cardiovascular diseases (CVD) and other comorbidities.

## Introduction

Obesity is an important risk factor contributing to the development of cardiovascular diseases (CVD) and increased mortality [1-2]. Worldwide, the prevalence of obesity has increased dramatically over the past three decades and is acknowledged as one of the most serious public health challenges of the 21st century [3]. If the excess body fat gained during young age persists during adulthood, there is an increased risk of developing chronic diseases, such as CVD and type-II diabetes, later in life [4]. Obesity results from a long-term energy imbalance, a combination of excess energy intake, and low levels of energy expenditure due to an inactive lifestyle [4-5].

The identification and prevention of obesity during young age is an important strategy to reduce present and future health risks. Creating a successful intervention that prevents or reduces obesity requires a better understanding of the lifestyle factors associated with obesity. In Nepal, data on the influence of lifestyle on obesity among youths are scarce, although this country is experiencing an epidemiologic transition [6]. There have been no publications in Nepal on the lifestyle factors associated with obesity among youths till now, making it difficult to develop an effective intervention. Information emerging from this study can fill this gap.

In this cross-sectional study, our aim was to determine the prevalence of obesity in youths and to explore the potential lifestyle factors among young university students (17–24 years) in Kathmandu City, Nepal.

## Materials and methods

A cross-sectional study was carried out at the Institute of Medicine (IOM), Tribhuvan University, in Kathmandu City. The study was conducted from 3rd March 2017 to 27th September 2017. The sample size of 384 undergraduate students aged 17-24, from either gender, was estimated using the Epi Info software developed by Centers for Disease Control and Prevention (CDC). It was calculated based on a population of 407,934 university students, 95% confidence interval, 5% acceptable margin of error, and the population proportion has been assumed to be 0.50, as this magnitude yielded the maximum possible sample size required [7]. The sampling frame was the list of undergraduate students from campuses affiliated to the Institute of Medicine, Tribhuvan University, and a simple random sampling method was used to select the sample. Participants were selected if they were willing to participate, were healthy and physically active, and took no medications known to influence fat metabolism. The study was approved by the Institutional Review Board (IRB), IOM. Study objectives were explained and written informed consent was obtained from participants prior to the study.

A self-administered questionnaire was designed by the investigators and information was collected about age, sex, smoking, alcohol consumption, meat consumption, fast-food consumption, and sedentary lifestyle. The questionnaire was assessed for content validity by a group of local experts. This consultation process led to redrafting and reorganizing items in the questionnaire. The questionnaire was pilot tested with five people, who were representative of the study population, to determine the clarity of the language used and the questionnaire structure. Some words were changed based on responses. Students who had smoked cigarettes within the last 30 days were defined as current smokers. Similarly, current episodic heavy alcohol consumption or binge drinking was defined, as five or more drinks of alcohol on at least one occasion on one or more of the 30 days preceding the survey. A sedentary lifestyle included those participants with less than 150 minutes of moderate physical activity or less than 60 minutes of vigorous physical activity per week [8]. Participants who had consumed fast food and meat within a week were defined as fast-food consumers and meat consumers, respectively.

Height was measured by a non-stretchable plastic tape after having the subject stand straight against an even wall. The body weight of all the subjects was measured using a standardized weighing machine, which was calibrated in kilograms. The body mass index (BMI) was calculated as weight in kg/height in square meters. The classification of BMI was as follows: 18.5-24.9 kg/m2 (normal), 25-29.9 kg/m2 (overweight), and more than 30.0 kg/m2 (obese). The waist circumference was measured using a non-stretchable plastic tape over the unclothed abdomen, at the umbilical level, in a standing position. Hip circumference was measured over light clothing at the widest point over the buttocks when viewed from the side. The waist-to-hip ratio was obtained by dividing the waist circumference by hip circumference. In accordance with WHO, a waist-to-hip ratio (WHR) above 0.90 for males and above 0.85 for females was considered obese [9].

Data was collected in Microsoft Excel (Ver. 2013) and statistical analysis was performed using SPSS 21 (IBM Corp., Version 21.0. Armonk, NY, US). Descriptive analyses (frequency and percentages) were used to characterize the samples. The independent t-test was used to compare the groups. A multiple regression analysis was employed to examine the predictors of BMI and WHR. The level of significance for acceptance was P < 0.05.

## Results

**Figure 1:**
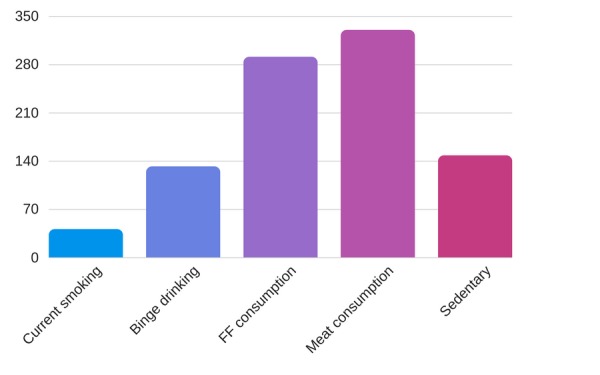
Descriptive statistics of lifestyle risk factors

Out of the 384 students who participated in our study, 41 (10.6 %) were current cigarette smokers while 132 (34.5%) were current episodic heavy alcohol consumers. Fast-food consumers and meat consumers were 291 (75.78%) and 330 (86 %), respectively. A sedentary lifestyle was present in 148 subjects (38.5 %). The descriptive statistics of various risk factors are illustrated in Figure 1. On the basis of BMI, out of the 384 students, 125 (32.5 %) were overweight and 44 (11.4%) were obese. Similarly, on the basis of WHR, 178 (46.35 %) were found to be obese.

This study inferred that binge drinking, cigarette smoking, and sedentary subjects had a statistically significant higher BMI and WHR. Subjects who consume meat had a statistically significantly higher BMI as well. However, fast-food consumers had no statistically significant difference in BMI and WHR as compared to those who don’t consume (Table 1). To test the independent influence of lifestyle parameters on BMI and WHR, we performed multiple linear regression analysis. Only binge drinking and cigarette smoking statistically significantly predicted BMI and WHR (Table 2). For every unit increase in binge drinking, a 1.44 unit increase in BMI and a 0.04 unit increase in WHR are predicted, holding all other variables constant. Similarly, for every unit increase in cigarette smoking, a 2.45 unit increase in BMI and a 0.03 unit increase in WHR are predicted, holding all other variables constant.

**Table 1:** Comparison of anthropometric parameters on the basis of various lifestyle parameters BMI: Body mass index WHR: Waist to hip ratio

Lifestyle parameters	BMI	P value	WHR	P value
Binge drinking				
Yes	23.30 ± 2.70	< 0.001	0.88 ± 0.04	< 0.001
No	20.90 ± 2.27		0.82 ± 0.06	
Cigarette smoking				
Yes	25.20 ± 2.41	< 0.001	0.90 ± 0.04	< 0.001
No	21.35 ± 2.45		0.84 ± 0.06	
Fast-food consumption				
Yes	21.65 ± 2.81	> 0.05	0.85 ± 0.05	> 0.05
No	21.60 ± 2.26		0.84 ± 0.06	
Meat consumption				
Yes	21.76 ± 2.64	< 0.05	0.84 ± 0.06	> 0.05
No	20.60 ± 2.24		0.84 ± 0.04	
Sedentary behavior				
Yes	22.10 ± 2.92	< 0.05	0.85 ± 0.06	< 0.05
No	21.26 ± 2.34		0.83 ± 0.06	

**Table 2:** Multiple linear regression analysis to test the independent influence of smoking and alcohol consumption on BMI and WHR BMI: Body mass index WHR: Waist to hip ratio

Predictor variable	Unstandardized coefficients		P value
	BMI (R ^2^ = 0.245)	WHR (R ^2 ^= 0.178)	
Cigarette smoking	2.45	0.03	< 0.001
Binge drinking	1.44	0.04	< 0.001

## Discussion

Obesity is a gain in body weight, conditioned by the accumulation of excessive body fat, significantly above the norms set for specific ages, races, and sexes, and exceeding the physiological needs and adaptability of the human body [10]. Anthropometric measurements are still popular for estimating the degree of body fattening. Among these, BMI is most frequently used, especially in population surveys [11]. Other variables, such as the WHR indicate abdominal fat distribution. These ratios can be useful for an evaluation of the risk of cardiovascular and metabolic diseases [12].

As indicated by the BMI, 125 (32.5 %) were overweight and 44 (11.4%) were obese; however, this figure increased to 178 (46.35 %) when WHR was considered a parameter of obesity. That means that central obesity is more prevalent than general obesity in our study population. The importance of the central distribution of fat has been known since decades. The INTERHEART study has shown that out of different anthropometric measures, the WHR shows the strongest relation with the risk of myocardial infarction. Moreover, this ratio was the strongest predictor of myocardial Infarction irrespective of age, sex, smoking status, diabetes, lipid level, and blood pressure [13]. The incidence of obesity, as per WHR, in our study is fairly high and merits the intervention for reducing abdominal circumference.

Our study found that current episodic heavy alcohol consumers had statistically significantly higher BMI and WHR. Wannamethee SG et al. found that in men, drinking > 21 drinks per week was associated with higher BMI or WHR compared to non-drinkers [14]. Based on the fact that 1 gram of alcohol provides 7.1 kcal (29 kJ) and studies showing that energy consumed as alcohol is additive to that from other dietary sources, increased energy intake with alcohol use can certainly promote a positive energy balance and ultimately weight gain [15]. Alcohol has also been shown to impact a number of hormones linked to satiety, such as inhibiting the effects of leptin [16].

A further observation from our study showed that current smokers had a statistically significant higher BMI and WHR. This was similar to the findings suggested by other standardized previous studies [17-18]. WHR is an indicator of the amount of visceral adipose tissue (VAT). Waist circumference is strongly associated with VAT and VAT is influenced by cortisol level [19]. Smokers have been shown to have higher fasting plasma cortisol levels than non-smokers [20-21]. Higher cortisol concentration could be the consequence of the stimulation of sympathetic nervous system activity that is induced by smoking [22-23]. Furthermore, an imbalance between estrogen and testosterone in females and a decrease in testosterone in males could play a role in the effect of smoking on visceral adipose tissue [24-25].

Another disclosure from our study was that meat consumers had a statistically significantly higher BMI, which was in accordance with an Indian study [26]. These are likely due to the high energy and fat content of meat dishes in the Indian subcontinent. According to the Nepal annual household survey 2014/15, the second-largest share in food expenditure is for ‘meat and fish’ (17.4%) [27]. Our finding of a slightly stronger association between meat consumption and generalized obesity than with central obesity assessed using WHR is interesting and might be due to the high protein content in meat as compared to fat.

Our analysis revealed that sedentary subjects had a statistically significant higher BMI and WHR. Experimental studies in humans have demonstrated that increased sedentary behavior is associated with reduced energy expenditure, the development of an insulin-resistance state, impaired insulin sensitivity, and an accumulation of abdominal fat [28]. Prolonged activities that do not require body mobility, such as computer usage and studying, are to the most extent unavoidable sedentary behaviors among university students. So, physical activity interventions like sports activities and establishing university gyms within campus premises can play a detrimental role to some extent.

Interestingly, fast-food consumers and nonconsumers had no statistically significant difference in BMI and WHR in our study. Eating in university cafeterias is very common among university students. Similarly, visits to fast-food restaurants are growing even more rapidly to the same extent. A recent Nepalese study reported that less than half of the food handlers had received training in food hygiene and safety, whereas trained food handlers exhibit negligence in maintaining hygienic conditions [29]. In the same study, out of 48 fast food samples examined, 39 were found contaminated and nine were free from any disease-causing microorganism, which may result in food poisoning, gastroenteritis, and other illnesses, leading to weight loss. Contamination of fast food may be the cause of no significant difference in BMI and WHR among fast-food consumers and nonconsumers in our study.

The main strength of the present study is the confirmation of a high prevalence of obesity among young university students of Nepal, making it necessary to develop and employ effective preventive measures. The findings will be useful to policy-makers, programme managers, and researchers in the design and implementation of interventions for the prevention and control of obesity in the youth population. This study has several limitations. First, we did not take genetic background into consideration. Second, the study design was limited to undergraduate medical and healthcare students. Third, self-administered questionnaires are susceptible to a recall bias. Additionally, because this was a cross-sectional study, causal relationships were not established. However, we maintained a standard procedure to measure BMI and WHR, and we applied a standard case definition to detect obesity.

## Conclusions

Our study shows a high prevalence of obesity among the young university students of Nepal, making it necessary to develop effective preventive measures. This study also indicated that smoking, drinking alcohol, meat consumption, and sedentary behavior are significantly associated with BMI and WHR. Early interventions to encourage more physical activity, decrease sedentary behavior, reduce alcohol consumption, abstain from smoking, and avail of balanced nutrition can be a strategy to prevent and/or reduce the risk of CVD development and other associated comorbidities, such as type 2 diabetes, stroke, hypertension, and dyslipidemia, among the youth.
